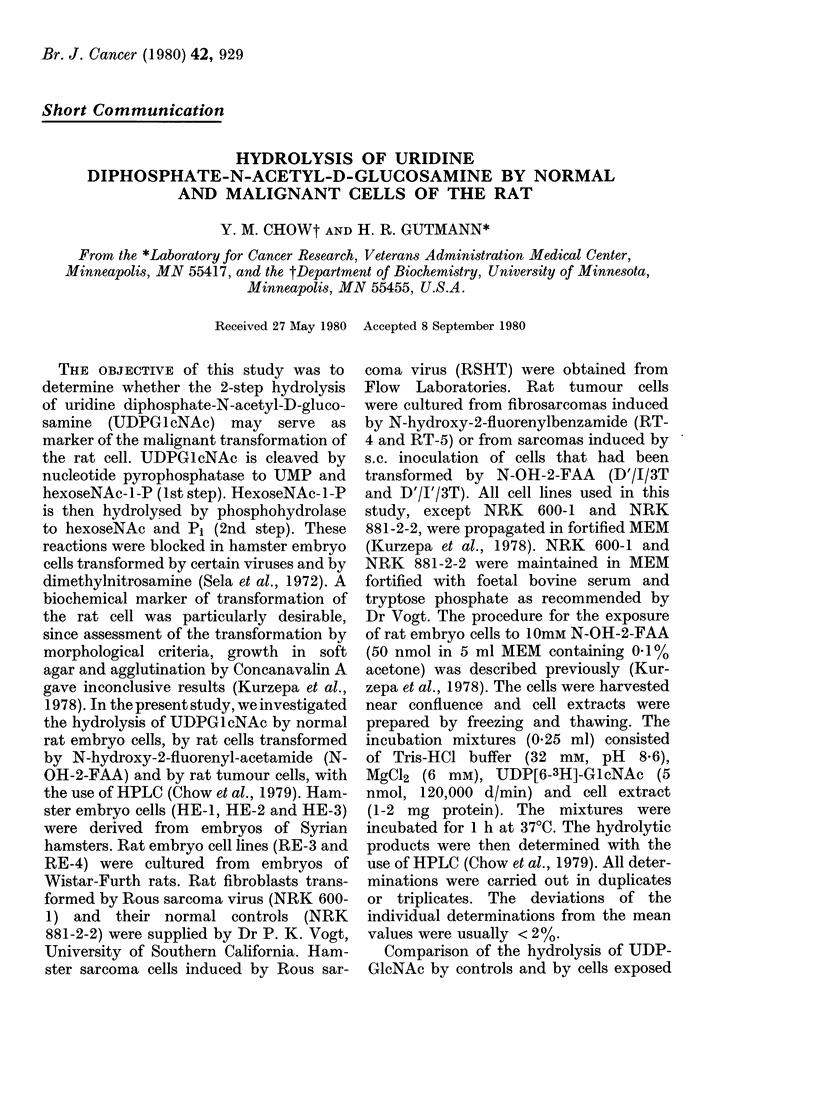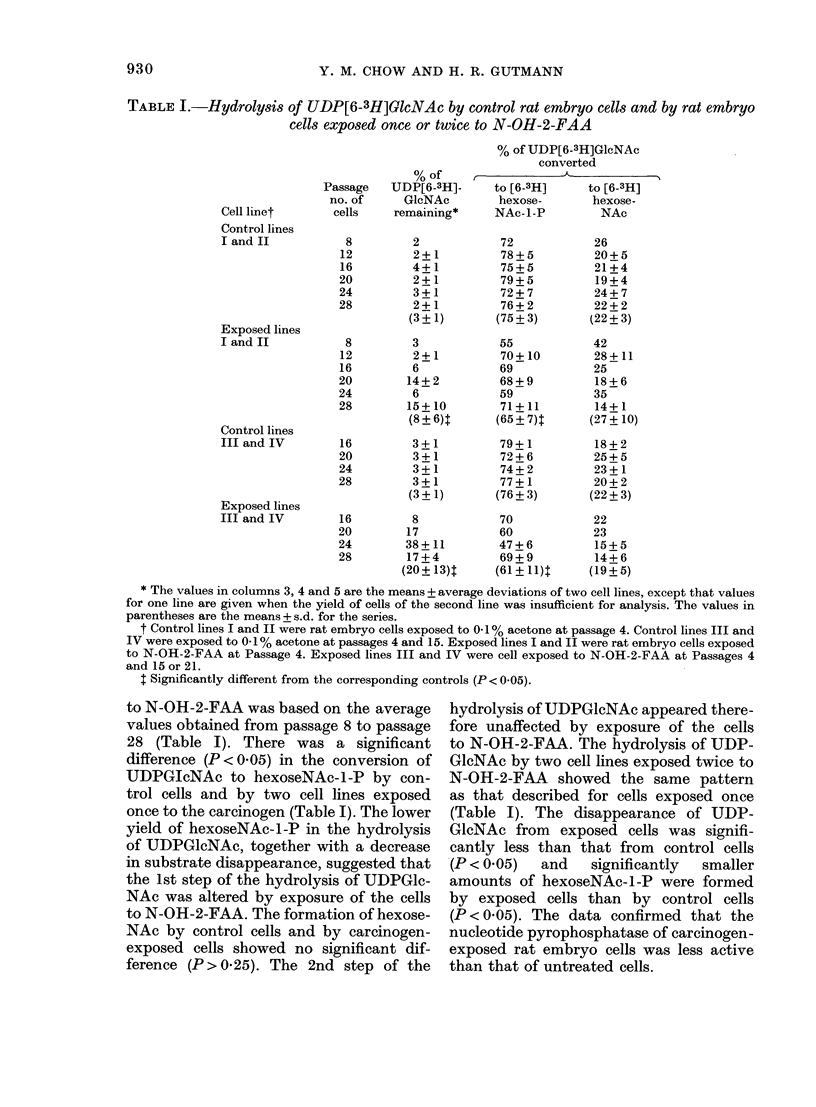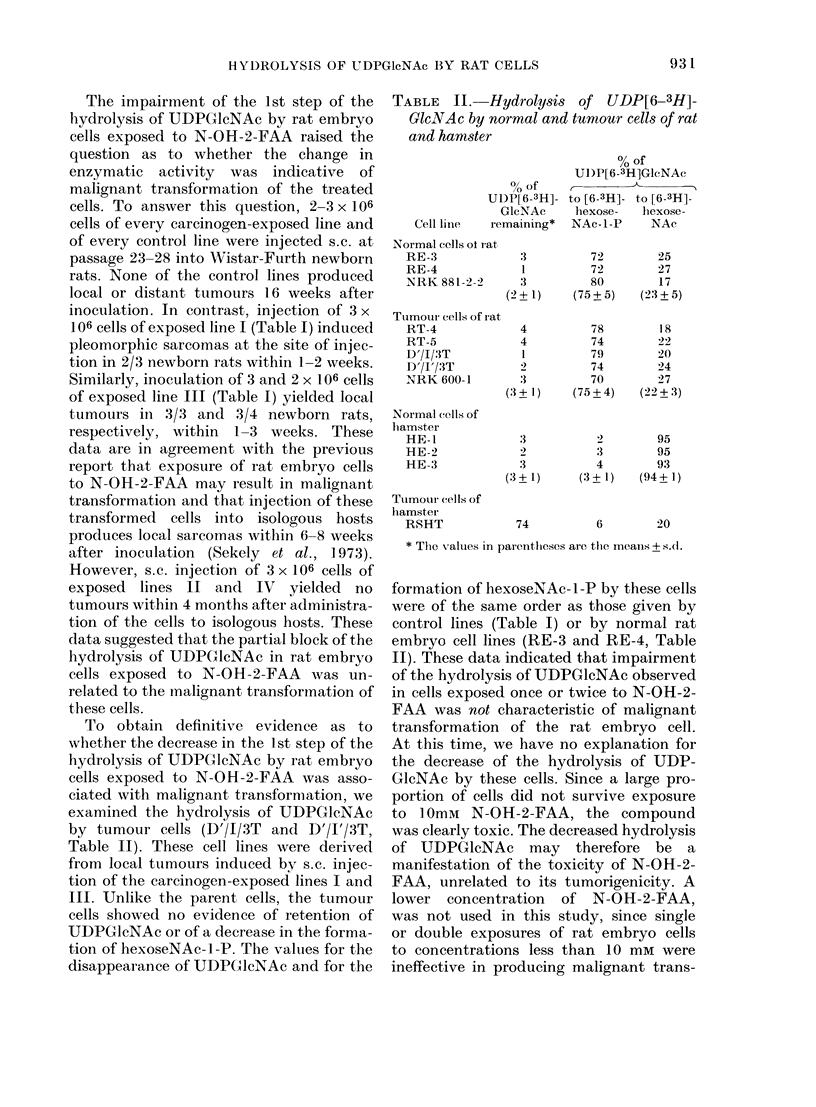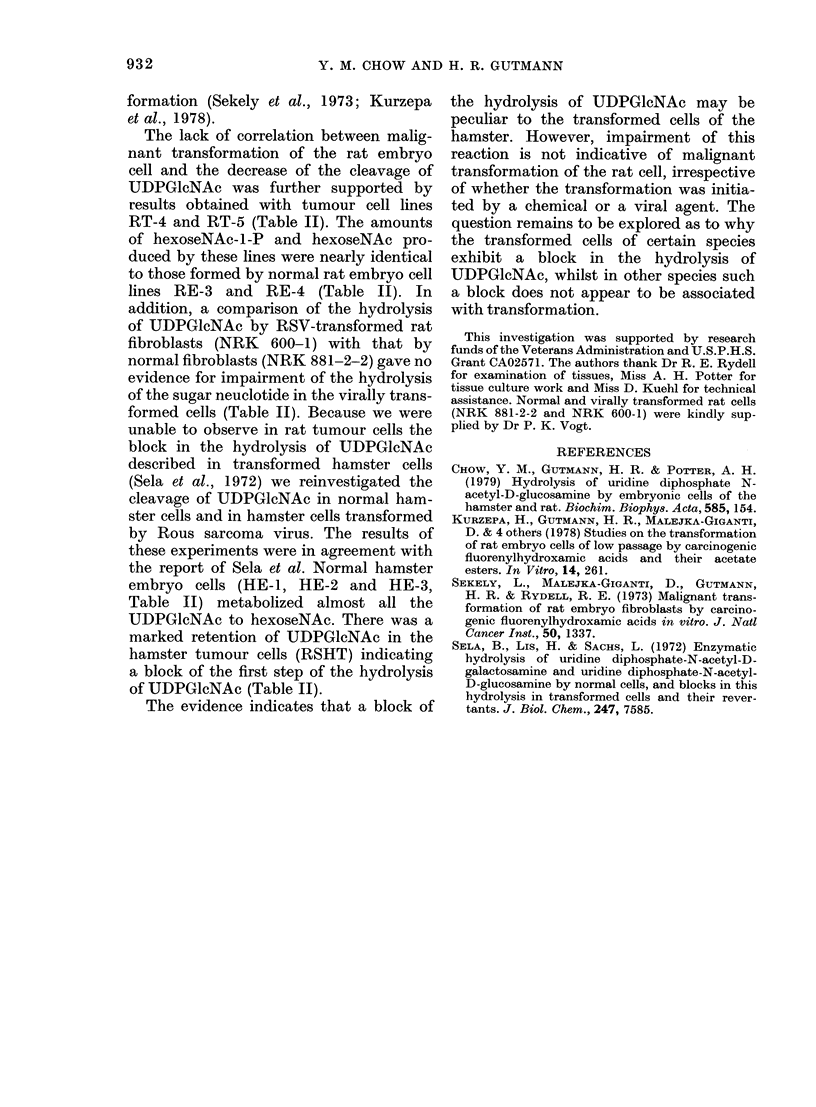# Hydrolysis of uridine diphosphate-N-acetyl-D-glucosamine by normal and malignant cells of the rat.

**DOI:** 10.1038/bjc.1980.342

**Published:** 1980-12

**Authors:** Y. M. Chow, H. R. Gutmann


*Br. J. Cancer* (1980) **42**, 929

***Short Communication***

### HYDROLYSIS OF URIDINE DIPHOSPHATE-N-ACETYL-D-GLUCOSAMINE BY NORMAL AND MALIGNANT CELLS OF THE RAT

Y. M. CHOW† AND H. R. GUTMANN*

*From the *Laboratory for Cancer Research, Veterans Administration Medical Center, Minneapolis, MN 55417, and the †Department of Biochemistry, University of Minnesota, Minneapolis, MN 55455, U.S.A.*

Received 27 May 1980 Accepted 8 September 1980

THE OBJECTIVE of this study was to determine whether the 2-step hydrolysis of uridine diphosphate-N-acetyl-D-glucosamine (UDPGlcNAc) may serve as marker of the malignant transformation of the rat cell. UDPGlcNAc is cleaved by nucleotide pyrophosphatase to UMP and hexoseNAc-1-P (1st step). HexoseNAc-1-P is then hydrolysed by phosphohydrolase to hexoseNAc and $P_i$ (2nd step). These reactions were blocked in hamster embryo cells transformed by certain viruses and by dimethylnitrosamine (Sela *et al.*, 1972). A biochemical marker of transformation of the rat cell was particularly desirable, since assessment of the transformation by morphological criteria, growth in soft agar and agglutination by Concanavalin A gave inconclusive results (Kurzepa *et al.*, 1978). In the present study, we investigated the hydrolysis of UDPGlcNAc by normal rat embryo cells, by rat cells transformed by N-hydroxy-2-fluorenyl-acetamide (N-OH-2-FAA) and by rat tumour cells, with the use of HPLC (Chow *et al.*, 1979). Hamster embryo cells (HE-1, HE-2 and HE-3) were derived from embryos of Syrian hamsters. Rat embryo cell lines (RE-3 and RE-4) were cultured from embryos of Wistar-Furth rats. Rat fibroblasts transformed by Rous sarcoma virus (NRK 600-1) and their normal controls (NRK 881-2-2) were supplied by Dr P. K. Vogt, University of Southern California. Hamster sarcoma cells induced by Rous sarcoma virus (RSHT) were obtained from Flow Laboratories. Rat tumour cells were cultured from fibrosarcomas induced by N-hydroxy-2-fluorenylbenzamide (RT-4 and RT-5) or from sarcomas induced by s.c. inoculation of cells that had been transformed by N-OH-2-FAA (D′/I/3T and D′/I′/3T). All cell lines used in this study, except NRK 600-1 and NRK 881-2-2, were propagated in fortified MEM (Kurzepa *et al.*, 1978). NRK 600-1 and NRK 881-2-2 were maintained in MEM fortified with foetal bovine serum and tryptose phosphate as recommended by Dr Vogt. The procedure for the exposure of rat embryo cells to 10mM N-OH-2-FAA (50 nmol in 5 ml MEM containing 0·1% acetone) was described previously (Kurzepa *et al.*, 1978). The cells were harvested near confluence and cell extracts were prepared by freezing and thawing. The incubation mixtures (0·25 ml) consisted of Tris-HCl buffer (32 mM, pH 8·6), $MgCl_2$ (6 mM), UDP[6-$^3$H]-GlcNAc (5 nmol, 120,000 d/min) and cell extract (1-2 mg protein). The mixtures were incubated for 1 h at 37°C. The hydrolytic products were then determined with the use of HPLC (Chow *et al.*, 1979). All determinations were carried out in duplicates or triplicates. The deviations of the individual determinations from the mean values were usually $< 2\%$.

Comparison of the hydrolysis of UDP-GlcNAc by controls and by cells exposed

TABLE I.—*Hydrolysis of UDP[6-³H]GlcNAc by control rat embryo cells and by rat embryo cells exposed once or twice to N-OH-2-FAA*

| Cell line† | Passage no. of cells | % of UDP[6-³H]-GlcNAc remaining* | % of UDP[6-³H]GlcNAc converted to [6-³H] hexose-NAc-1-P | % of UDP[6-³H]GlcNAc converted to [6-³H] hexose-NAc |
|---|---|---|---|---|
| Control lines I and II | 8 | 2 | 72 | 26 |
| | 12 | $2\pm1$ | $78\pm5$ | $20\pm5$ |
| | 16 | $4\pm1$ | $75\pm5$ | $21\pm4$ |
| | 20 | $2\pm1$ | $79\pm5$ | $19\pm4$ |
| | 24 | $3\pm1$ | $72\pm7$ | $24\pm7$ |
| | 28 | $2\pm1$ | $76\pm2$ | $22\pm2$ |
| | | $(3\pm1)$ | $(75\pm3)$ | $(22\pm3)$ |
| Exposed lines I and II | 8 | 3 | 55 | 42 |
| | 12 | $2\pm1$ | $70\pm10$ | $28\pm11$ |
| | 16 | 6 | 69 | 25 |
| | 20 | $14\pm2$ | $68\pm9$ | $18\pm6$ |
| | 24 | 6 | 59 | 35 |
| | 28 | $15\pm10$ | $71\pm11$ | $14\pm1$ |
| | | $(8\pm6)$‡ | $(65\pm7)$‡ | $(27\pm10)$ |
| Control lines III and IV | 16 | $3\pm1$ | $79\pm1$ | $18\pm2$ |
| | 20 | $3\pm1$ | $72\pm6$ | $25\pm5$ |
| | 24 | $3\pm1$ | $74\pm2$ | $23\pm1$ |
| | 28 | $3\pm1$ | $77\pm1$ | $20\pm2$ |
| | | $(3\pm1)$ | $(76\pm3)$ | $(22\pm3)$ |
| Exposed lines III and IV | 16 | 8 | 70 | 22 |
| | 20 | 17 | 60 | 23 |
| | 24 | $38\pm11$ | $47\pm6$ | $15\pm5$ |
| | 28 | $17\pm4$ | $69\pm9$ | $14\pm6$ |
| | | $(20\pm13)$‡ | $(61\pm11)$‡ | $(19\pm5)$ |

* The values in columns 3, 4 and 5 are the means ± average deviations of two cell lines, except that values for one line are given when the yield of cells of the second line was insufficient for analysis. The values in parentheses are the means ± s.d. for the series.

† Control lines I and II were rat embryo cells exposed to 0·1% acetone at passage 4. Control lines III and IV were exposed to 0·1% acetone at passages 4 and 15. Exposed lines I and II were rat embryo cells exposed to N-OH-2-FAA at Passage 4. Exposed lines III and IV were cell exposed to N-OH-2-FAA at Passages 4 and 15 or 21.

‡ Significantly different from the corresponding controls ($P < 0{\cdot}05$).

to N-OH-2-FAA was based on the average values obtained from passage 8 to passage 28 (Table I). There was a significant difference ($P < 0{\cdot}05$) in the conversion of UDPGIcNAc to hexoseNAc-1-P by control cells and by two cell lines exposed once to the carcinogen (Table I). The lower yield of hexoseNAc-1-P in the hydrolysis of UDPGlcNAc, together with a decrease in substrate disappearance, suggested that the 1st step of the hydrolysis of UDPGlc-NAc was altered by exposure of the cells to N-OH-2-FAA. The formation of hexose-NAc by control cells and by carcinogen-exposed cells showed no significant difference ($P > 0{\cdot}25$). The 2nd step of the hydrolysis of UDPGlcNAc appeared therefore unaffected by exposure of the cells to N-OH-2-FAA. The hydrolysis of UDP-GlcNAc by two cell lines exposed twice to N-OH-2-FAA showed the same pattern as that described for cells exposed once (Table I). The disappearance of UDP-GlcNAc from exposed cells was significantly less than that from control cells ($P < 0{\cdot}05$) and significantly smaller amounts of hexoseNAc-1-P were formed by exposed cells than by control cells ($P < 0{\cdot}05$). The data confirmed that the nucleotide pyrophosphatase of carcinogen-exposed rat embryo cells was less active than that of untreated cells.

The impairment of the 1st step of the hydrolysis of UDPGlcNAc by rat embryo cells exposed to N-OH-2-FAA raised the question as to whether the change in enzymatic activity was indicative of malignant transformation of the treated cells. To answer this question, $2–3 \times 10^6$ cells of every carcinogen-exposed line and of every control line were injected s.c. at passage 23–28 into Wistar-Furth newborn rats. None of the control lines produced local or distant tumours 16 weeks after inoculation. In contrast, injection of $3 \times 10^6$ cells of exposed line I (Table I) induced pleomorphic sarcomas at the site of injection in 2/3 newborn rats within 1–2 weeks. Similarly, inoculation of 3 and $2 \times 10^6$ cells of exposed line III (Table I) yielded local tumours in 3/3 and 3/4 newborn rats, respectively, within 1–3 weeks. These data are in agreement with the previous report that exposure of rat embryo cells to N-OH-2-FAA may result in malignant transformation and that injection of these transformed cells into isologous hosts produces local sarcomas within 6–8 weeks after inoculation (Sekely *et al.*, 1973). However, s.c. injection of $3 \times 10^6$ cells of exposed lines II and IV yielded no tumours within 4 months after administration of the cells to isologous hosts. These data suggested that the partial block of the hydrolysis of UDPGlcNAc in rat embryo cells exposed to N-OH-2-FAA was unrelated to the malignant transformation of these cells.

To obtain definitive evidence as to whether the decrease in the 1st step of the hydrolysis of UDPGlcNAc by rat embryo cells exposed to N-OH-2-FAA was associated with malignant transformation, we examined the hydrolysis of UDPGlcNAc by tumour cells (D′/I/3T and D′/I′/3T, Table II). These cell lines were derived from local tumours induced by s.c. injection of the carcinogen-exposed lines I and III. Unlike the parent cells, the tumour cells showed no evidence of retention of UDPGlcNAc or of a decrease in the formation of hexoseNAc-1-P. The values for the disappearance of UDPGlcNAc and for the formation of hexoseNAc-1-P by these cells were of the same order as those given by control lines (Table I) or by normal rat embryo cell lines (RE-3 and RE-4, Table II). These data indicated that impairment of the hydrolysis of UDPGlcNAc observed in cells exposed once or twice to N-OH-2-FAA was *not* characteristic of malignant transformation of the rat embryo cell. At this time, we have no explanation for the decrease of the hydrolysis of UDPGlcNAc by these cells. Since a large proportion of cells did not survive exposure to 10mM N-OH-2-FAA, the compound was clearly toxic. The decreased hydrolysis of UDPGlcNAc may therefore be a manifestation of the toxicity of N-OH-2-FAA, unrelated to its tumorigenicity. A lower concentration of N-OH-2-FAA, was not used in this study, since single or double exposures of rat embryo cells to concentrations less than 10 mM were ineffective in producing malignant trans-

TABLE II.—*Hydrolysis of UDP[6–³H]-GlcNAc by normal and tumour cells of rat and hamster*

| Cell line | % of UDP[6-³H]-GlcNAc remaining* | % of UDP[6-³H]GlcNAc to [6-³H]-hexose-NAc-1-P | % of UDP[6-³H]GlcNAc to [6-³H]-hexose-NAc |
|---|---|---|---|
| Normal cells of rat | | | |
| RE-3 | 3 | 72 | 25 |
| RE-4 | 1 | 72 | 27 |
| NRK 881-2-2 | 3 | 80 | 17 |
| | (2 ± 1) | (75 ± 5) | (23 ± 5) |
| Tumour cells of rat | | | |
| RT-4 | 4 | 78 | 18 |
| RT-5 | 4 | 74 | 22 |
| D′/I/3T | 1 | 79 | 20 |
| D′/I′/3T | 2 | 74 | 24 |
| NRK 600-1 | 3 | 70 | 27 |
| | (3 ± 1) | (75 ± 4) | (22 ± 3) |
| Normal cells of hamster | | | |
| HE-1 | 3 | 2 | 95 |
| HE-2 | 2 | 3 | 95 |
| HE-3 | 3 | 4 | 93 |
| | (3 ± 1) | (3 ± 1) | (94 ± 1) |
| Tumour cells of hamster | | | |
| RSHT | 74 | 6 | 20 |

* The values in parentheses are the means ± s.d.

formation (Sekely *et al.*, 1973; Kurzepa *et al.*, 1978).

The lack of correlation between malignant transformation of the rat embryo cell and the decrease of the cleavage of UDPGlcNAc was further supported by results obtained with tumour cell lines RT-4 and RT-5 (Table II). The amounts of hexoseNAc-1-P and hexoseNAc produced by these lines were nearly identical to those formed by normal rat embryo cell lines RE-3 and RE-4 (Table II). In addition, a comparison of the hydrolysis of UDPGlcNAc by RSV-transformed rat fibroblasts (NRK 600–1) with that by normal fibroblasts (NRK 881–2–2) gave no evidence for impairment of the hydrolysis of the sugar neuclotide in the virally transformed cells (Table II). Because we were unable to observe in rat tumour cells the block in the hydrolysis of UDPGlcNAc described in transformed hamster cells (Sela *et al.*, 1972) we reinvestigated the cleavage of UDPGlcNAc in normal hamster cells and in hamster cells transformed by Rous sarcoma virus. The results of these experiments were in agreement with the report of Sela *et al.* Normal hamster embryo cells (HE-1, HE-2 and HE-3, Table II) metabolized almost all the UDPGlcNAc to hexoseNAc. There was a marked retention of UDPGlcNAc in the hamster tumour cells (RSHT) indicating a block of the first step of the hydrolysis of UDPGlcNAc (Table II).

The evidence indicates that a block of the hydrolysis of UDPGlcNAc may be peculiar to the transformed cells of the hamster. However, impairment of this reaction is not indicative of malignant transformation of the rat cell, irrespective of whether the transformation was initiated by a chemical or a viral agent. The question remains to be explored as to why the transformed cells of certain species exhibit a block in the hydrolysis of UDPGlcNAc, whilst in other species such a block does not appear to be associated with transformation.

This investigation was supported by research funds of the Veterans Administration and U.S.P.H.S. Grant CA02571. The authors thank Dr R. E. Rydell for examination of tissues, Miss A. H. Potter for tissue culture work and Miss D. Kuehl for technical assistance. Normal and virally transformed rat cells (NRK 881-2-2 and NRK 600-1) were kindly supplied by Dr P. K. Vogt.

### REFERENCES

CHOW, Y. M., GUTMANN, H. R. & POTTER, A. H. (1979) Hydrolysis of uridine diphosphate N-acetyl-D-glucosamine by embryonic cells of the hamster and rat. *Biochim. Biophys. Acta*, **585**, 154.

KURZEPA, H., GUTMANN, H. R., MALEJKA-GIGANTI, D. & 4 others (1978) Studies on the transformation of rat embryo cells of low passage by carcinogenic fluorenylhydroxamic acids and their acetate esters. *In Vitro*, **14**, 261.

SEKELY, L., MALEJKA-GIGANTI, D., GUTMANN, H. R. & RYDELL, R. E. (1973) Malignant transformation of rat embryo fibroblasts by carcinogenic fluorenylhydroxamic acids *in vitro*. *J. Natl Cancer Inst.*, **50**, 1337.

SELA, B., LIS, H. & SACHS, L. (1972) Enzymatic hydrolysis of uridine diphosphate-N-acetyl-D-galactosamine and uridine diphosphate-N-acetyl-D-glucosamine by normal cells, and blocks in this hydrolysis in transformed cells and their revertants. *J. Biol. Chem.*, **247**, 7585.